# Radiographic grid for locating foreign bodies in maxillofacial emergency trauma

**DOI:** 10.1186/s12903-023-03807-0

**Published:** 2024-01-08

**Authors:** Ziqi Zhang, Mingliang Yang, Ran Zhang

**Affiliations:** Department of Oral and Maxillofacial Surgery, School and Hospital of Stomatology, Liaoning Provincial Key Laboratory of Oral Diseases, China Medical University, Shenyang, 110002 China

**Keywords:** Foreign bodies, Radiography, Radiographic grid, CT

## Abstract

**Objectives:**

The accurate localization of the foreign bodies (FBs) is essential. This work presents a new noninvasive technique for subcutaneous metallic FBs under a radiographic grid, a system that simplifies the localization of facial FBs removal using a grid with embedded reference points.

**Methods:**

This work designed a retrospective study to evaluate the effect of a radiographic grid on FBs removal surgery. All patients who met the inclusion criteria and attended the Hospital of Stomatology of China Medical University from January 2022 to June 2023 were enrolled and randomly divided into grid and non-grid groups. The assessment of facial swelling, the primary indicator, was conducted on days 2 and 7 post-surgery. The variables were analyzed using the Student t test and a repeated-measures general linear model.

**Results:**

The study sample consisted of 20 patients, with 14 males (70%) and 6 females (30%), who had an average age of 30.30 ± 5.38. The average time of operation was 1.85 ± 0.66 h (range 0.7 to 3.2). In the present cases in this report, of the 20 patients’ FBs, 14 were metal, 5 were glass, and 1 was residual root. And the FBs were surgically removed with no postoperative complications. Through comparison, it was found that the degree of swelling on day 2 postoperatively was significantly different between the grid group and the non-grid group (*P* < 0.05).

**Conclusions:**

This study demonstrates that a radiographic grid with mark points is a more efficient approach compared with traditional methods for FBs removal, and this surgical method is more accurate, fast and noninvasive.

## Introduction

Foreign bodies (FBs) are the most common accompanying injury in patients with maxillofacial trauma [1]. FBs are defined as anything that comes from outside the body. Commonly encountered FBs include wood, glass, plastic, or retained metals [2]. Numerous studies have indicated that around one-third of all FBs go unnoticed initially, often being discovered incidentally on radiographs. This oversight can give rise to substantial complications [3, 4]. These FBs can cause irritation, inflammation, infection, abscess formation, pain, swelling, migration, and potential damage to blood vessels or nerves. In addition, inflammatory reactions and the development of granulomas can impede wound healing. In some cases, FBs may even lead to severe complications like intracranial abscesses. To prevent complications, it is crucial to detect and remove FBs as soon as possible [5, 6].

Accurate localization of foreign bodies (FBs) is crucial, especially when they are located in critical areas such as near large vessels and the removal procedure poses a high risk to the patient’s safety [7]. Therefore, precise diagnosis and localization of FBs are of utmost importance due to the complex anatomical features of the maxillofacial and neck regions. Blind exploration can further damage the tissue and push the FBs deeper into the fascial planes [8]. Moreover, sharp FBs may migrate over time towards vital structures, leading to severe complications [9]. The patient’s medical history and clinical and radiological examinations are essential for diagnosing and localizing the FB [10].

Traditional radiography, ultrasonography (US), computerized tomography (CT), magnetic resonance imaging (MRI), and fluoroscopy are different diagnostic tools used to locate FBs [11]. However, accurately determining the exact position of an object in soft tissue using these techniques can be challenging. To overcome these obstacles and to help locate and remove FBs in maxillofacial emergencies, this work presents a new noninvasive technique for subcutaneous metallic FBs under a radiographic grid, a system that simplifies the localization of facial FBs removal using a grid with embedded reference points.

## Materials and methods

This work designed a retrospective study to evaluate the effect of a radiographic grid on FBs removal surgery. The inclusion criteria for the study consisted of patients presenting to the emergency department with a history of metallic and impalpable subcutaneous foreign bodies lodged in their body. All patients who met the inclusion criteria and attended the Hospital of Stomatology of China Medical University from January 2022 to June 2023 were enrolled and randomly divided into grid and non-grid groups by random number table. The following exclusion criteria were used: FBs are too small or the risk of removal is too large.; FBs are transparent to X-rays; pericoronitis or infection; systemic conditions; recent administration of anti-inflammatory medications or antibiotics; allergic history; pregnancy or breastfeeding; cysts or tumors in the maxillofacial region; and patients who were lost in the follow-up. A comprehensive medical history was collected and the patients underwent a thorough clinical examination. The affected area was carefully examined by palpation. The primary variable for evaluating facial swelling was measured on postoperative days 2 and 7.

**Fig. 1:**
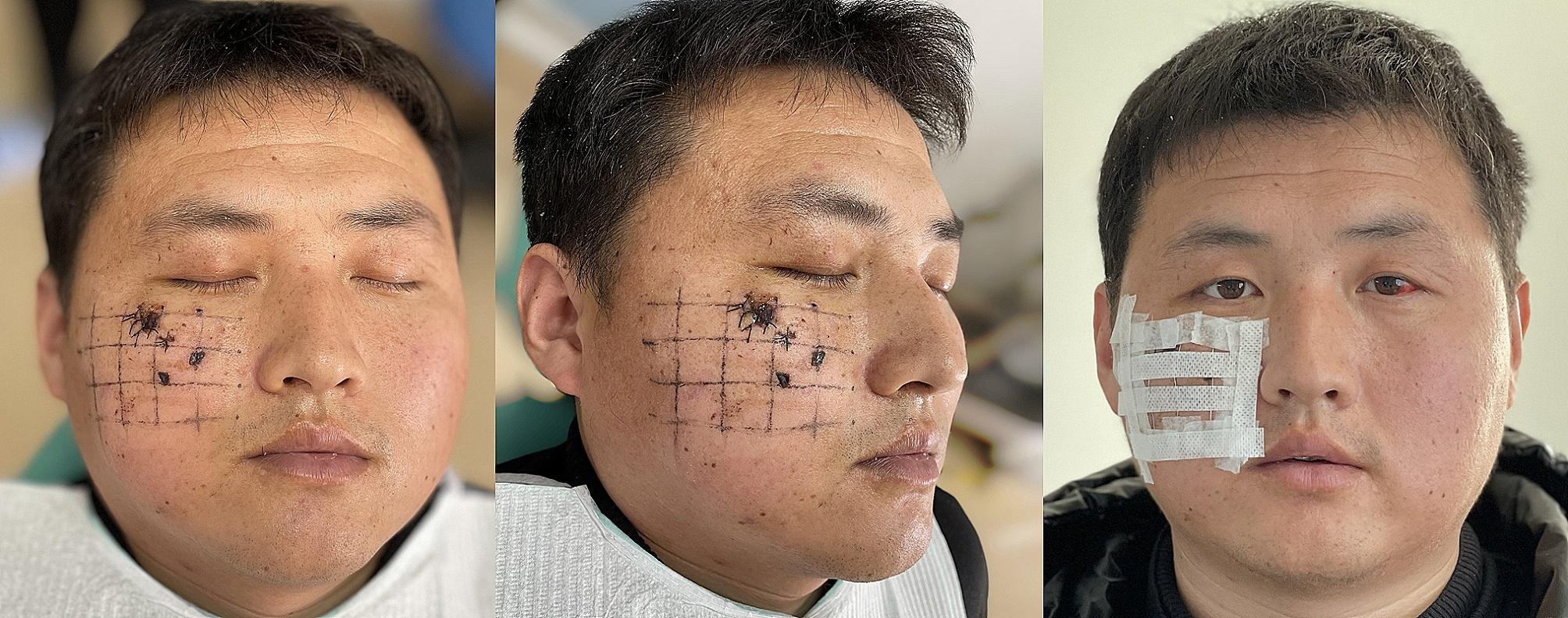
Before CT scan, a reference grid was made with disposable angiographic catheters according to size and position of FBs, which was taped in parallel alignment at fixed intervals. The grid is placed over the skin, and accurate CT are obtained

A disposable angiographic catheter offers the advantage of having radiopacity and causing no metal artifacts. Prior to the CT scan, a disposable angiographic catheters were used to create a reference grid, which was then securely taped in parallel alignment at regular intervals. The grid was made corresponding to the FBs according to their size and position. The grid is placed over the skin, and accurate CT are obtained (Fig. 1). All study subjects’ MSCT data were acquired using the TOSHIBA X-ray high voltage machine (Aquilion TSX-101 A), with a voxel size of 0.3 mm. Scans were conducted at 50 mA and 120 kV, and reconstructed at 0.3 mm before being exported in DICOM format. A single photographer completed all MSCT scans. After CT scan, Digital Imaging and Communications in Medicine (DICOM) data was imported into Proplan software to perform the analysis. The Proplan software is utilized to process and edit anatomical data obtained from acquired imaging data. It enables the creation of 3D designs, 3D surface models, and 3D measurements. By employing post-processing reconstructions and adjusting the window settings, the software can enhance the diagnosis of challenging-to-locate foreign bodies based on factors such as size, density, and orientation.

**Fig. 2:**
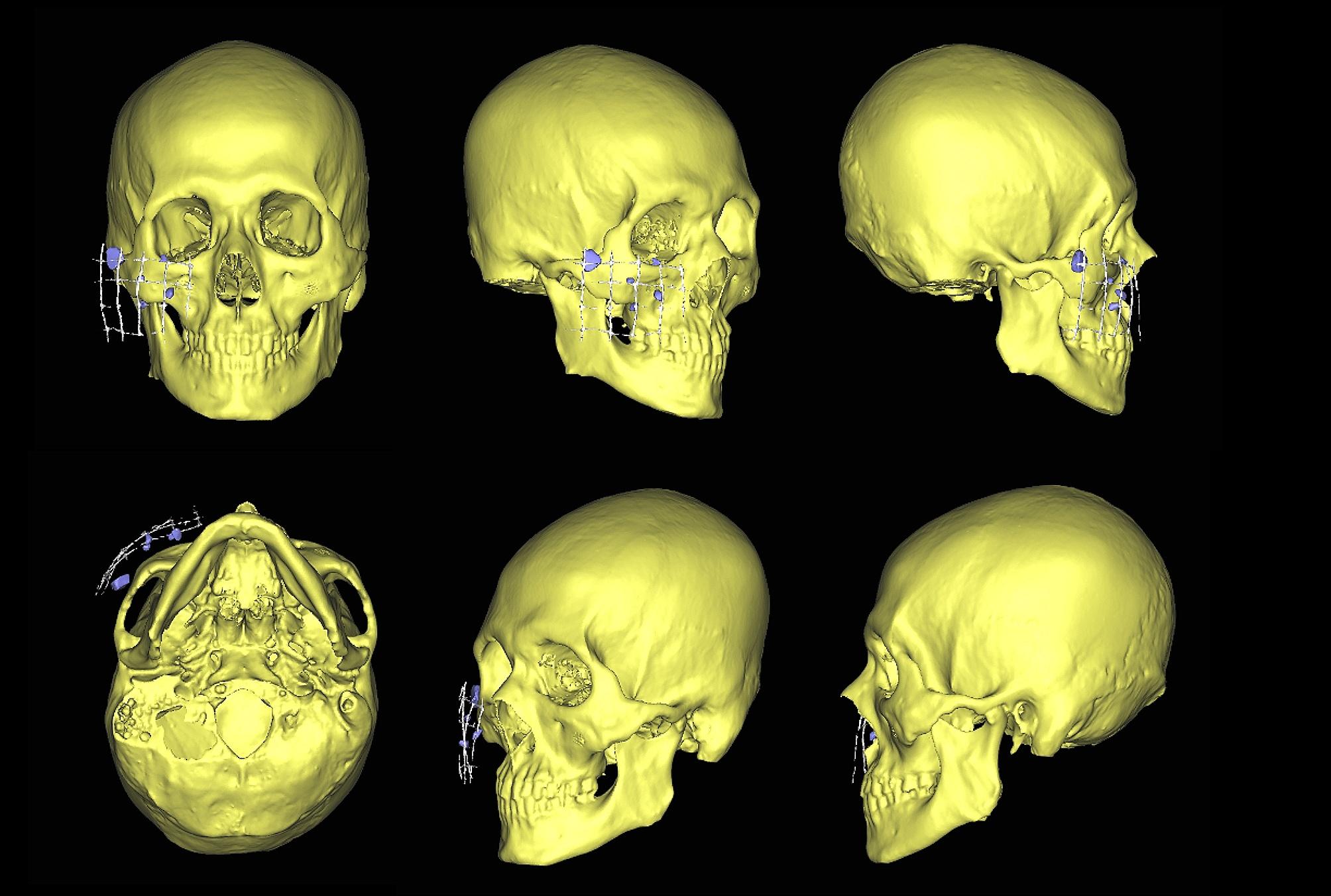
After CT scan, DICOM data was imported into Proplan software to perform the analysis. Segment FBs, reference grids and bone and create 3D model

**Fig. 3:**
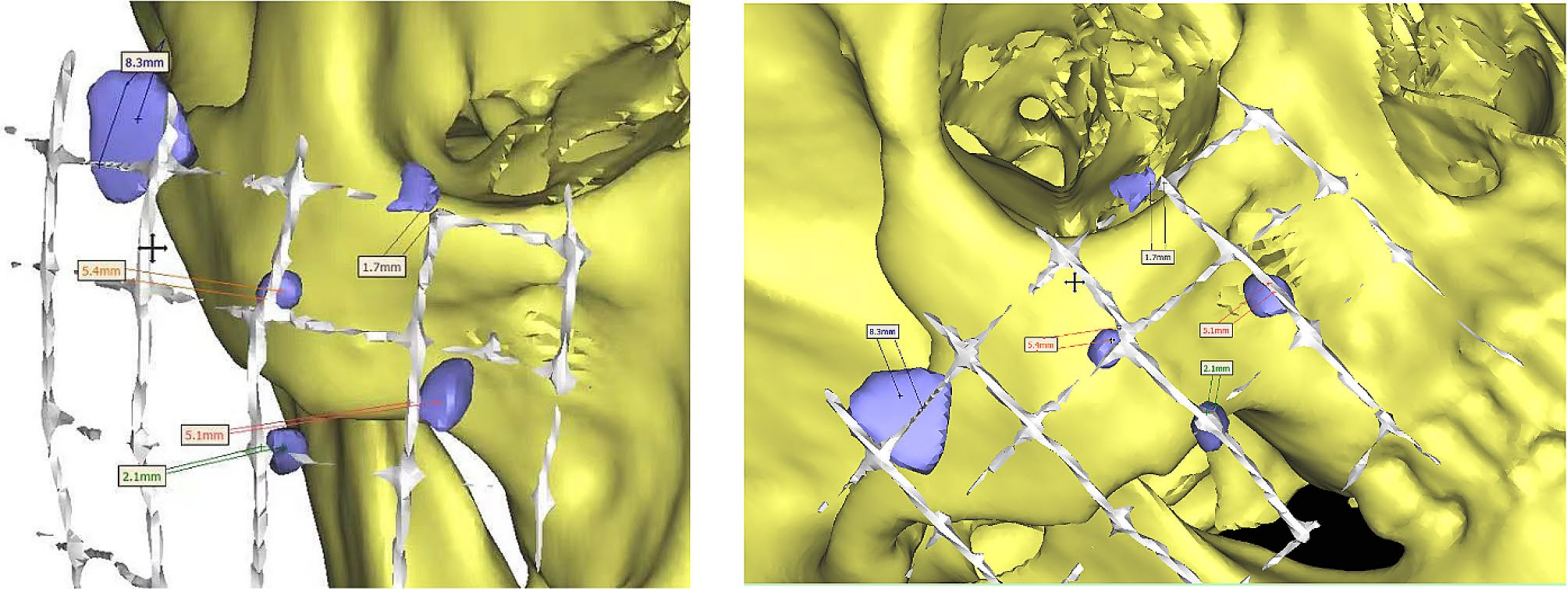
Measure the distance between the FBs and each grid marker or anatomic marker

The XYZ axes were used to properly orient the image. A customized threshold value was employed to create a new mask for the FBs. During the multiple-slice editing phase, the FBs were segmented and separated from the surrounding structures slice by slice (Fig. 2). Afterwards, the broken segment underwent region-growing in the software. Once the region was successfully grown, the 3D model of the FBs was generated. Measure the distance between the foreign body and each grid marker or anatomic marker (Fig. 3).

**Fig. 4:**
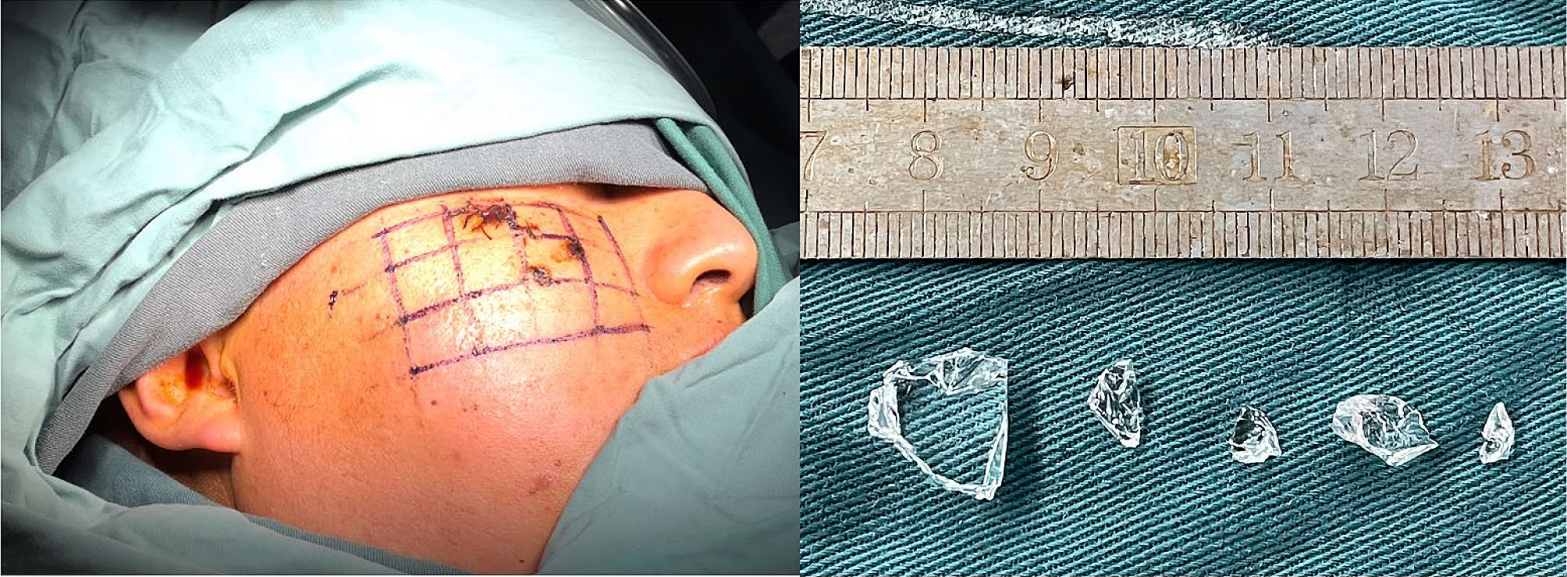
Mark the skin along the mesh with a skin marker and carefully remove the mesh. (left) FBs removed from this surgery.(right)

After determining the FB site, a small skin incision was made with a local anesthetic administered. The length of the incision was adjusted based on the shape and size of the FB. However, the length should be at least as long as the object’s shortest dimension (the width) in order to minimize tissue damage while allowing for a smooth and natural extraction of the object. Marking the skin along the mesh with a skin marker and carefully remove the mesh. The marked area nearer to the foreign body was then explored. Proceed to the next grid after adequate exploration of one grid to ensure removal of all FBs and to reduce bleeding and migration of FBs. Identification of maxillofacial FBs and remove them (Fig. 4). Finally, rinsing the wound, closing the incision, administering antibiotics to prevent infection, and administering Tetanus prophylaxis.

All surgical procedures were performed under general anesthesia and carried out by the same oral surgeon. This retrospective study has been approved by the Institutional ethics committee of School and Hospital of Stomatology, China Medical University (No. k2021038). The study design adheres to the ethical principles outlined in the Declaration of Helsinki for medical research involving human subjects. Prior to study participation, informed consent was obtained from the patient’s parents or legal guardians.

A modified tape-measure technique was utilized to determine the extent of postoperative swelling, as described by Gabka and Matsumura [12]. At 2 and 7 days after the surgery, three measurements were taken from the following 5 reference points: soft tissue pogonion, angle of the mandible, tragus, outer corner of the mouth, and lateral corner of the eye. The contralateral measurements were taken as the baseline for evaluating the respective side of the face. The average of each postoperative measurement and the corresponding average baseline value were used to assess the level of facial edema on a specific day.

## Results

The study sample consisted of 20 patients, with 14 males (70%) and 6 females (30%), who had an average age of 30.30 ± 5.38 (ranging from 19 to 39 years old). The average time of operation was 1.85 ± 0.66 h (range 0.7 to 3.2). In the present cases in this report, of the 20 patients’ FBs, 14 were metal, 5 were glass, and 1 was residual root. And the FBs were surgically removed with no postoperative complications (Table 1).

**Table 1:** Demographic characteristics of patients

Case	Group	Gender	Age	Type of FBs	Number	Time of operation(h)
1	Grid group	F	19	metal	solitary	3.2
2	Grid group	M	28	glass	multiple	2.8
3	Grid group	M	25	glass	multiple	1.8
4	Grid group	F	24	residual root	solitary	0.7
5	Grid group	M	33	glass	multiple	1.1
6	Grid group	M	32	metal	solitary	0.9
7	Grid group	F	23	metal	solitary	1.3
8	Grid group	M	34	glass	multiple	2.5
9	Grid group	F	26	metal	solitary	1.8
10	Grid group	F	35	metal	multiple	1.9
11	Non-grid group	M	36	metal	solitary	1.5
12	Non-grid group	M	26	metal	solitary	1.3
13	Non-grid group	M	38	metal	solitary	1.4
14	Non-grid group	M	39	metal	solitary	2.1
15	Non-grid group	M	31	metal	solitary	2.2
16	Non-grid group	M	32	metal	multiple	2.7
17	Non-grid group	F	28	metal	solitary	2.1
18	Non-grid group	M	29	metal	multiple	1.5
19	Non-grid group	M	32	glass	multiple	1.9
20	Non-grid group	M	36	metal	solitary	2.3

For grid group patients, on day 2 postoperatively, the average swelling was 15.3 mm on the affected side compared to 14.2 mm on the healthy side. The difference in swelling between the groups on the second day after surgery was statistically significant. (*P* < 0.05). For non-grid group patients, on day 2 postoperatively, the mean amount of swelling was 15.7 mm in the affected side versus 14.4 mm in the healthy side. The difference in swelling between the groups on the 2nd day after the surgery was found to be statistically significant (*P* < 0.05). On the 7th day after the surgery, it was observed that the affected side had a reduced swelling compared to the healthy side. However, the difference in swelling between the groups on the 7th day after the surgery was not statistically significant (*P* > 0.05) (Table 2).

**Table 2:** Comparison of facial swelling among different groups

	grid group			Non-grid group			
	Affected side(mm)	Healthy side(mm)	Differences	Affected side(mm)	Healthy side(mm)	Differences	*P* value*
2 days postoperatively	15.3 ± 0.9	14.2 ± 0.9	1.0 ± 0.4	15.7 ± 0.8	14.4 ± 0.7	1.3 ± 0.3	0.047
7 days postoperatively	14.9 ± 0.8	14.2 ± 0.9	0.8 ± 0.3	15.4 ± 0.8	14.4 ± 0.7	1.0 ± 0.3	0.190
*P* value+	0.005	0.678	0.008	0.000	1.000	0.001	

Note: The data are presented as mean ± standard deviation. A *P*-value of less than 0.05 is regarded as statistically significant

* Student t test

^+^ Repeated-measures general linear model

This work defined the difference between the amount of swelling on the affected side and the healthy side as the degree of swelling of the patient. Through comparison, it was found that the degree of swelling on day 2 postoperatively was significantly different between the grid group and the non-grid group (*P* < 0.05) (Table 2).

## Discussion

FBs account for approximately 3.8% of all pathological findings in the head and neck region [5, 13, 14]. The composition and placement of FBs may differ depending on the nature of the trauma. Common types of foreign bodies embedded in the soft tissues include wood, glass, metal, tooth, plastic, stone, and organic materials. Prompt identification and removal of impacted FBs are crucial to prevent potential complications including pain, inflammation, infection, damage to peripheral nerves or blood vessels, migration to adjacent regions, impaired wound healing due to granuloma formation, pseudoaneurysms, cellulitis, and abscess formation [15, 16]. FBs located near tendons can cause lesions and affect tendon movement. FBs near nerves can also lead to nerve lesions and pain, while FBs near blood vessels can cause thrombus formation [17]. In some cases, asymptomatic objects may remain embedded in the tissues. Deeply embedded splinters can be difficult to detect during a physical examination if there are few signs of a wound, inflammation, mass, or tenderness. Approximately one-third of foreign body impaction cases are missed during the initial examination due to their small size and/or deep location[18]. The type and size of the object, as well as its proximity to vital structures in the body, determine the surgical difficulty in retrieving it and the potential complications. Ensuring precise localization is essential for minimizing surgical complications, particularly in cases involving deeply embedded or closely located FBs near vital structures [14, 16]. It is important to promptly attempt the removal of a foreign body with minimal damage.

To the best of our knowledge, no previous study has been documented in the literature that assesses the impact of a radiographic grid surgical guide on the extraction of FBs in the maxillofacial region. As a result, our study is the pioneering research of its kind. The present investigation defines the degree of swelling in patients as the disparity in swelling between the affected and healthy sides. By comparing the two groups, the edema in the grid group was found to be significantly lower than that in the non-grid group, as observed 2 days post-operation.

Surgical removal of foreign bodies from the jaws can be challenging and time-consuming unless their precise location is known prior to the operation. Locating small, isolated bone lesions that are both radiopaque and radiolucent can be equally difficult in the jaws. Random surgical exploration in the bone can be frustrating and unproductive, sometimes even jeopardizing important anatomical structures. Various imaging techniques, including plain radiographs, ultrasound, CT, MRI, and fluoroscopy, have been utilized for diagnostic purposes.

The initial evaluation involves acquiring a detailed medical history, conducting a clinical examination, and performing routine radiography. Panoramic radiography is commonly employed as the primary imaging modality for detecting foreign bodies in the maxillofacial region [5, 16].

However, a significant portion (33%) of FBs, especially impermeable ones, are often missed during initial examination because conventional radiography cannot accurately display small permeable FBs, especially those located deeper within the body due to the overlapping of two-dimensional views and shadows of similar opacities [5, 13, 15, 18]. Plain radiographs have a success rate of detecting metallic FBs ranging from 69 to 90% and glass FBs from 71 to 77%, but there is limited or no information available regarding the identification of organic materials like wood (0 to 15%) [19].

Numerous studies have suggested CT as the preferred imaging technique for detecting FBs in the maxillofacial region due to its ability to differentiate substances based on Hounsfield Unit (HU) values, accurately locate FBs, and reconstruct their shape and size, assisting in the surgical removal of FBs [5, 10, 20]. However, CT scanning has limitations, such as high radiation exposure and considerable metal artifacts, posing a specific challenge in the detection of small metallic objects [5, 18, 21, 22]. In comparison, Cone Beam CT (CBCT) has a lower cost and radiation exposure. Therefore, CBCT imaging technology is preferred for suspected maxillofacial cases involving FBs [18]. A study assessed the diagnostic accuracy of CT and CBCT in identifying various FBs including metals, teeth, wood, plastics, glass stones, and graphite. They found that CT and CBCT imaging were able to detect all FBs, except for wood [16].

Ultrasonography is a cost-effective and convenient imaging technique that provides real-time visualization using non-radiative ultrasound. It is particularly effective in displaying objects in surface tissue, but not in areas near air, deep tissue, or behind bone structures [5, 13, 16].

MRI, with its high soft tissue contrast and multiplanar imaging capability, is a noninvasive modality commonly used. However, the use of MRI should be avoided if a metallic foreign body is suspected, as the magnetic field can mobilize metallic structures [19]. Therefore, MRI is absolutely contraindicated as a first-line evaluation for unknown foreign bodies. Additionally, the exorbitant expenses, lengthy procedures, susceptibility to subjective interpretations, and subpar precision constrain the utilization of MRI scans in individuals who have suffered dentoalveolar injuries [18, 20].

Navigation was introduced in the field of head and neck surgery over 20 years ago. While it was initially developed for neurosurgical purposes, its applications have expanded and gained recognition in maxillofacial surgery as well [23]. However, the usage of navigation systems in the mandible region is not authorized due to the continuous motion in this specific area.

To our knowledge, there has been no previous report on the use of a radiographic grid surgical guide for removing FBs in the maxillofacial region. In the past few years, there has been a growing popularity of 3D techniques using CBCT or CT images. These techniques have proven to be valuable in enhancing surgical preparation and serving as useful aids during surgery. However, it is worth noting that the preoperative planning needed for navigation surgery and 3D-printed assisted surgery can be time-consuming due to the planning and printing processes involved.

When the combination of a radiographic grid and 3D reconstruction is utilized, the precision of locating foreign bodies (FBs) and providing surgical guidance is surpassed only by navigation. However, this method requires only a CT machine, a wire, and a computer with Proplan software. Hence, the primary advantage highlighted by this study is that it is straightforward, convenient, and demands minimal equipment. Consequently, it can be widely implemented across hospitals of various levels, ensuring enhanced benefits for a larger number of patients.

In this case study, the use of a radiographic grid surgical guide proved to be effective in accurately determining the precise location, resulting in minimal yet adequate surgical access. This approach achieved a noninvasive procedure with fewer complications, potentially saving time and costs. It is crucial to minimize the time gap between preoperative imaging and the actual operation in order to mitigate the potential risk of migration [24–26]. If there is suspicion of foreign body movement, reassessment is necessary. The advantage of the technique employed in this study lies in the consistent relationship between the marker used to locate the grid and the reference frame, which represents the position of the patient’s facial feature and remains unchanged.

Another important aspect to ensure precision is the patient’s facial state and position while undergoing CT scanning: it should closely resemble the position required during surgery. The variation in the locations of FBs across different positions, as well as the discrepancy in FBs locations based on the patient’s positioning, may result in misdiagnosis and inappropriate surgical intervention. If an opened-mouth surgery or a closed-mouth surgery is planned, CT should be performed at the same posture. To ensure the optimal surgical outcome, excessive stretching of soft tissue during surgery should be avoided. A thorough preoperative discussion on CT imaging is crucial for surgical planning and anticipating potential challenges. Managing complex cases involving foreign bodies requires interdisciplinary team collaboration and coordination.

The choice of wire is also critical. The material and thickness of the steel wire should be such that they do not create artifacts during the CT scan, ensuring more accurate FB localization. Similarly, grid planning should also be taken into account. The size and position of the grid, as well as the number of reference points, will influence the localization of FBs.

Careful planning and meticulous scanning of the radiographic grid fairly localize most of the radioopaque FBs and aids easy removal as happened in our study. However, there are still certain limitations to this technique. One such limitation is the slice thickness of the CT scanner. FBs must exceed the minimum slice thickness for detection and precise localization. Another limitation of this study is the lack of discussion of foreign body size and the effect of foreign body size on surgical time and degree of swelling due to small sample size. And the time of surgery itself can affect the swelling. In future studies, the researchers will expand the sample size to further study the influence of various factors on postoperative recovery. Moreover, considering the multiple planning stages involved, the resultant object location may not be entirely precise. Hence, surgical skill, anatomical knowledge, and experience are of utmost importance. In the future, if this method can be integrated with navigation or engineering, the localization of FBs will be more precise.

## Conclusions

Precise identification of the exact location of FBs is crucial for safe removal without complications. Preoperative CT scanning and 3D image reconstruction can aid in accurately locating the object and provide a comprehensive view of the surrounding anatomy, including critical blood vessels. This study demonstrates that a radiographic grid with mark points is a more efficient approach compared with traditional methods for FBs removal, and this surgical method is more accurate, fast and noninvasive.

## Data Availability

All data generated or analysed during this study are included in this published article.
